# Real-Time Thermal Modulation of High Bandwidth MOX Gas Sensors for Mobile Robot Applications †

**DOI:** 10.3390/s19051180

**Published:** 2019-03-08

**Authors:** Yuxin Xing, Timothy A. Vincent, Marina Cole, Julian W. Gardner

**Affiliations:** School of Engineering, University of Warwick, Coventry CV4 7AL, UK

**Keywords:** MOX, mobile robot, thermal modulation, high-bandwidth, interactive mapping

## Abstract

A new signal processing technique has been developed for resistive metal oxide (MOX) gas sensors to enable high-bandwidth measurements and enhanced selectivity at PPM levels (<5 PPM VOCs). An embedded micro-heater is thermally pulsed from a temperature of 225 to 350 °C, which enables the chemical reaction kinetics of the sensing film to be extracted using a fast Fourier transform. Signal processing is performed in real-time using a low-cost microcontroller integrated into a sensor module. Three sensors, coated with SnO_2_, WO_3_ and NiO respectively, were operated and processed at the same time. This approach enables the removal of long-term baseline drift and is more resilient to changes in ambient temperature. It also greatly reduced the measurement time from ~10 s to 2 s or less. Bench-top experimental results are presented for 0 to 200 ppm of acetone, and 0 ppm to 500 ppm of ethanol. Our results demonstrate our sensor system can be used on a mobile robot for real-time gas sensing.

## 1. Introduction

Robotic systems have been of interest in many fields, such as manufacturing [1], medical health care [2], military [3], agriculture [4], and also in emergency response [5]. They are ideal tools for exploring hazardous areas without placing the lives of emergency responders (e.g., firefighters) at risk. A mobile robot can assess the environment prior to the deployment of human teams. With its help, the potential risks to personnel can be reduced and hidden dangers eliminated, allowing firefighters to have a safer working environment. In a disaster zone, such as a burning building, visibility can be poor. The robot requires sensors other than machine vision to navigate, such as LIDAR and RADAR [6], in order to generate real-time maps and images with detailed information. Such equipment is vital for fire and rescue teams. The robot is equipped with imaging systems and thus can be teleoperated in low-visibility conditions (e.g., smoke-filled or low-light enclosed areas) or it can autonomously navigate to a designated safe location. A multi-sensor unit is also required, particularly in areas with possible gas contaminations or unknown gas substances, for gas discrimination, localization, mappings and comprehensive environmental monitoring. 

Many common gas sensors for environmental monitoring are unsuitable for robot applications. Commercial gas sensors, often employ technologies such as electrochemical, acoustic, catalytic or semiconductor [7], are generally bulky with slow response and poor selectivity. They can be sensitive to ambient temperature or background noise (acoustic sensors), prone to poisoning in harsh conditions (catalytic sensors), and fragile to use. For sensors to be used on a mobile exploration robot, it has to be robust, resilient, and at the same time, with high sensitivity and fast response. In this work, CMOS based nanostructured metal oxide sensors (MOX) are used. A customized metal oxide coating is deposited on top of a commercially available micro-hotplate, which enhanced the sensor’s sensitivity and selectivity. Three MOX sensors are housed inside a metal enclosure to protect them from any physical damages. The unit is then installed on a mobile robot used to create a hazard map of the surrounding areas. The map is generated in real-time from the sensor outputs and displayed to the user via an interactive screen. Locations of gas sources and a map of the gas concentration can be overlaid onto the map. 

The sensing mechanism of the MOX sensor is based on the interaction between the chemisorbed oxygen site in the metal oxide layer and the target gas. In an n-type semiconductor MOX sensor (e.g., SnO_2_), electrons are generated and the depletion region decreases when the gas is introduced, causing a decrease in resistivity. For a p-type sensor, the accumulation region increases with the gas, causing an increase in resistivity. The MOX gas sensor is widely used commercially due to its balanced overall performance, high sensitivity and low fabrication cost [8]. They can be commonly found in air quality monitoring for VOC detections. However, commercial sensors have the disadvantage of a slow response time (>30 s [9]), long stabilization period before measurements (~24 h), and longer-term performance drift. This is not ideal for our application as a fast response is crucial. To compensate those flaws, we demonstrated a novel thermal modulation signal processing technique by using the ‘dynamic’ response of MOX sensors. 

In ‘static’ mode, sensors are operated with fixed current, hence fixed temperature, which is typically above 100 °C but below 500 °C [10]. In the ‘dynamic’ mode, sensors are operated between two temperature settings. As MOX sensors are much more sensitive towards temperature changes, by switching the micro-hotplate ‘ON’ (higher temperature) and ‘OFF’ (lower temperature), we are able to remove baseline drift, decrease response time and reduce power consumption. By doing so, those high-bandwidth sensors can respond in 2 s or possibly even less without any stabilization period beforehand. This technique has been previously reported [11,12] as a technique to enhance MOX sensor performance. However, studies to implement this technique in real time on robots for instant indications are limited. Here we configured the temperature modulation algorithms on a commercial Teensy 3.6 microcontroller, which produces a user-friendly output in real-time on the mobile robot. Our preliminary results have been presented in EUROSENSORS 2018 conference [13], where one MOX sensor result was used as an example to proof of concept. In this work, three MOX sensors are used simultaneously with the outputs processed in real-time on the microcontroller. This output can then be used just by itself for fast sensors response, or to replace the raw signal data presented in [14] for a more accurate gas mapping. This unit was also presented in a previously published paper where the ‘static’ mode of the sensors was used in a customized wind tunnel for plumes investigation [15]. 

## 2. Sensing Unit

**Figure 1:**
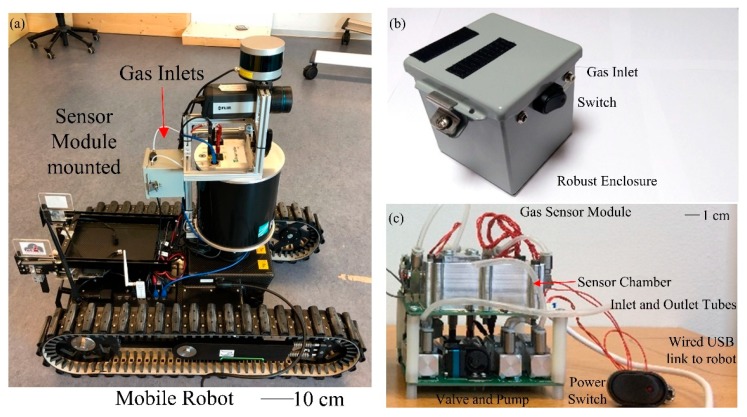
Photographs showing robust gas sensor module, (**a**) module installed on tracked exploration robot, (**b**) steel enclosure holding sensor module with inlet for gas sampling, and (**c**) internal components of the sensor module, stack PCB design, upper board contains low power sensor components, and lower board contains flow control circuitry (pump, valves and flow sensor).

Equipment designed for use by firefighter agencies must be able to withstand extreme temperatures for a given period of time as experienced during a typical rescue operation. A gas sensing unit, housed inside a robust enclosure, was designed to be integrated on a mobile robot provided by Taurob (Austria), shown in Figure 1a, forms the mobile exploration platform for hazardous environments. The unit containing three MOX sensors with Pt/Pd doped n-type tin dioxide (SnO_2_), tungsten trioxide (WO_3_) and p-type nickel oxide (NiO) film coatings, respectively. The unit has a size of 10 cm × 10 cm × 10 cm steel enclosure to protect the sensors and electronic components from falling debris and high temperatures. Figure 1b shows the outer view of the unit and Figure 1c shows the interior. This compact two-layer stacked Printed Circuit Board (PCB) design has the sensor chamber and microcontroller on the top, and mechanical component for gas sampling system (pump, valves, and flow sensor) on the bottom. Two gas sampling inlets are used, in a physically high and low position, to enable gases that have a lower or greater density than air to be sampled. A filter is placed before the sensors to prevent particulate poisoning of the chemical sensors. It has been reported that fire-fighters can encounter room temperatures >400 °C when tackling a fire inside a building [16]. 

The compact unit is shielded from direct contact with the extreme temperatures through the use of a heat shield, which can be deployed to cover the robot (not shown). 

**Figure 2:**
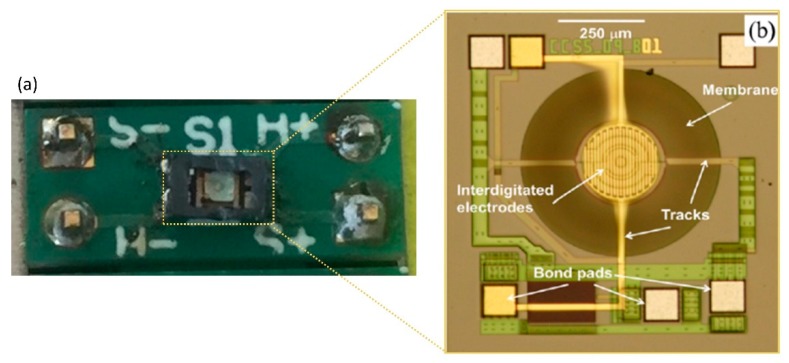
Photographs of a metal oxide device, (**a**) daughter for MOX sensor with DFN (dual-flat no-leads) package (marked with dotted line) and (**b**) microscopic image of the bare micro-hotplate, provided by ams Sensors Ltd (Cambridge, UK).

The three MOX devices are based on commercial silicon on insulator (SOI) micro-hotplates [17], which is commercially available from ams Sensors UK Ltd (bare chip shown in Figure 2b). The sensor has a size of 1 mm × 1 mm, which is bonded to a DFN package then soldered onto a daughter board (Figure 2a) for easy replacement. The customized coating was developed in house and deposited via a manual drop coating technique. As stated above two n-type (SnO_2_ and WO_3_) and one p-type (NiO) devices were used. The characteristics of SnO_2_ and WO_3_ coated MOX sensors are presented in [18] and [10], which show their high sensitivity towards CO and NO_2_. However, for safety reasons, the hazardous gases are replaced with innocuous gases/vapors such as acetone and ethanol in this work. 

**Figure 3:**
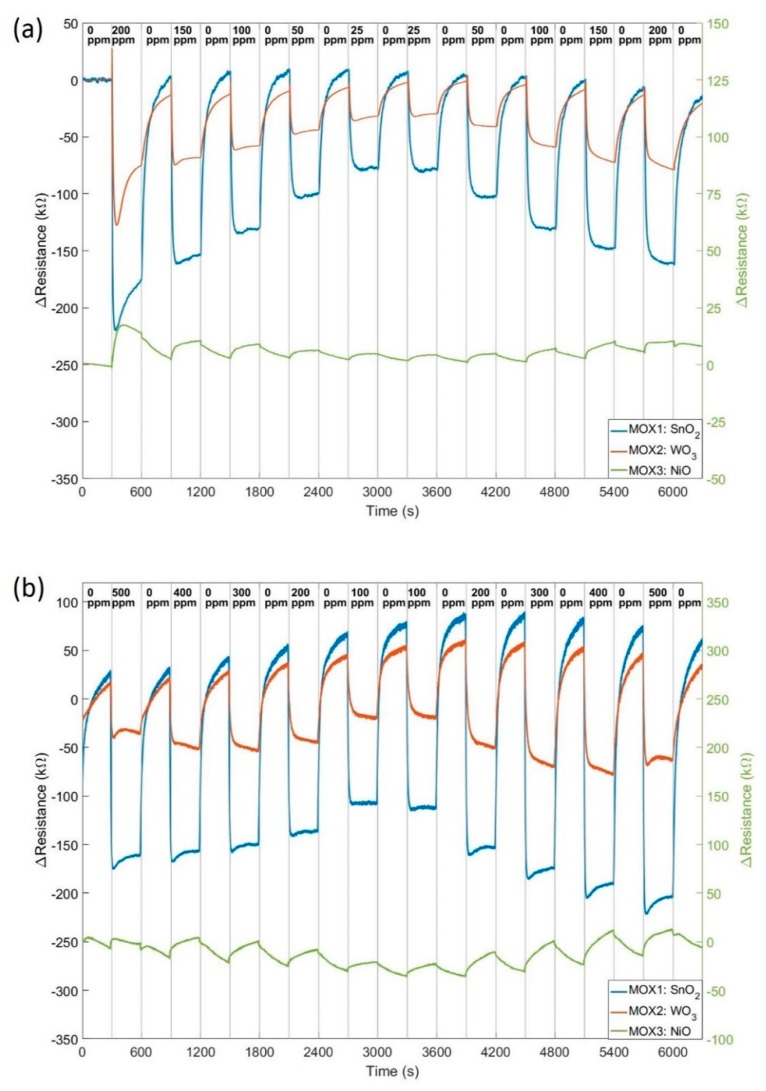
Change of resistances of three MOX sensors (Pt/Pd doped SnO_2_, WO_3_ and NiO) towards (**a**) acetone from 25 ppm to 200 ppm in 5 min steps, and (**b**) ethanol from 100 ppm to 500 ppm in 5 min steps. Synthetic air baseline between gases, both under dry condition.

The sensors were first tested in ‘static’ DC mode for characterization. The sensitivity and response speed of the MOX sensor increases with operation temperature, but high temperature reduces the lifetime. So initial tested were performed to find the balance between sensitivity and lifetime. The optimum temperature was found at around 350 °C, which with this miniature micro-hotplate, has less than 75 mW power consumption per sensor. The sensors are driven by an adjustable constant current circuitry with the Teensy microcontroller sets the current value. The unit was connected to a gas testing bench for measurements. Gas concentrations from 25 to 200 ppm of acetone and 100 ppm to 500 ppm ethanol were trialed with five minutes steps and synthetic air baseline in between. Sensors were driven by a Teensy microcontroller with data visualized to LabVIEW at 10 Hz sampling rate and stored to an onboard microSD card in 100 Hz. Figure 3 shows the changes in resistance of three MOX sensors in acetone (Figure 3a) and ethanol (Figure 3b). 

This results demonstrated our novel high bandwidth sensors offer a good sensitivity to low ppm levels. The ‘static’ response time is 10 s, which is superior to MOX sensors currently available on the market. The data were post-processed with a filter to remove drift. Even though, the baseline between each concentration is slightly different each time. This requires a longer stabilization period between measurements, which is not realistic for a mobile robot in emergency response. As the micro-hotplate has a fast switching time (10 ms for the rise and 30 ms for the fall) and MOX sensors are more sensitive to temperature changes, we applied a ‘dynamic’ mode to our sensors to improve the response speed.

## 3. Thermal Modulation

### 3.1. Method

**Figure 4:**
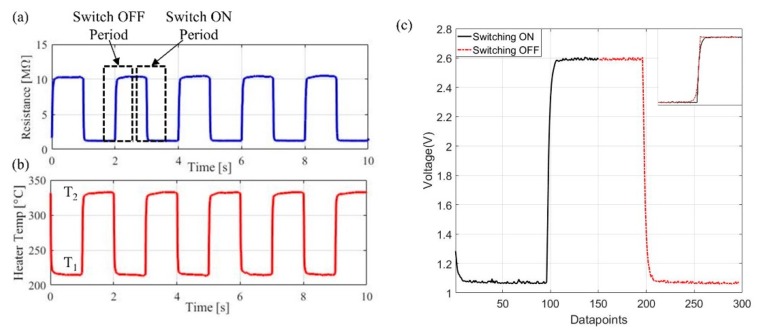
Steps signal processing method used during thermal modulation, the sensor is thermally modulated between two temperatures. (**a**) Change in resistance of a thermally modulated MOX (reference coating used, resistance magnitude does not correspond to gas measurements) and heater temperature shown in (**b**), and (**c**) one step of the raw MOX sensor during measurement, with black line as ‘switching ON’ period and red as ‘switching OFF’ period.

In the ‘dynamic’ operation, the microheater is pulsed between two temperatures to switch ‘ON’ and ‘OFF’. The miniature sensors then uniquely offer the possibility to extract data during the transition period between those two temperatures. In Reference [19], the authors introduced the temperature modulated MOX sensors with heaters switching between various temperature ranges, from 200 °C to 350 °C. They found out higher temperature and bigger temperature steps have the best results. So we pulsed the heater between 225 °C (‘OFF’) and 325 °C (‘ON’), which generates an average power consumption of <60 mW. The pulse period is 2 s with 1 s at each temperature level. Figure 4 shows the sensor response during thermal modulation. Figure 4a,b is the resistance changes of the sensor and microheater temperature changes. 

**Figure 5:**
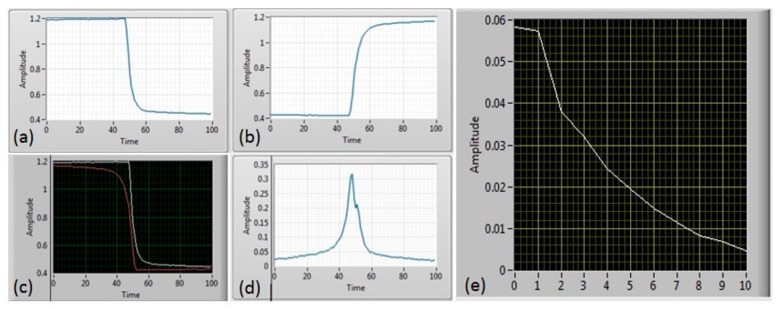
Thermal modulation steps visualized in LabVIEW. One sensor pulse is used as an example, the falling (**a**) and rising (**b**) part flipped (**c**), and subtracted (**d**). Results are then processed through FFT to get amplitude readings (**e**).

One set of data is extracted from each switching cycle, as shown in Figure 4c. Readings are taken at both temperatures and subtracted, thus removing the effect of signal drift (affected by environmental temperature). Considering the conductance changes, G, of the sensor in quasi-steady-state, as the sensor in gas at a constant concentration, the output from the sensor can be represented via:

(1) $$G_{1}\left(t\right)=G_{T1}+\left(G_{T2}-G_{T1}\right)\;\left[1-exp\;\left(-t/\tau_{1}\right)\right]$$

(2) $$G_{2}\left(t\right)=G_{T2}-\left(G_{T2}-G_{T1}\right)\;\left[1-exp\;\left(-t/\tau_{2}\right)\right],$$

where $\tau_{1}$ and $\tau_{2}$ are the rise and fall time of the conductance, *T*_1_ and *T*_2_ are the ‘OFF’ and ‘ON’ temperature, as marked in Figure 4b. Equation (1) represents the transition period increasing in temperature (switch ‘ON’ period) and Equation (2) represents the period when the temperature is decreased (switch ‘OFF’ period). These periods are represented as exponential changes. To calculate the response of the sensor to the target gas, remove drift and eliminate the change in resistance due to heater temperature, the ‘OFF’ period (Equation (2)) is flipped (as shown in the zoom in view in Figure 4c) and subtracted from the ‘ON’ period (Equation (1)). A fast Fourier transform (FFT) is then performed on the subtracted data (Figure 5d) to find the peak magnitude, which is different for gases at different concentration levels. Those steps have been performed offline in MATLAB initially before implemented into Teensy 3.6 microcontroller for real time data output. LabVIEW program is used to visualize the steps as shown in Figure 5. 

**Figure 6:**
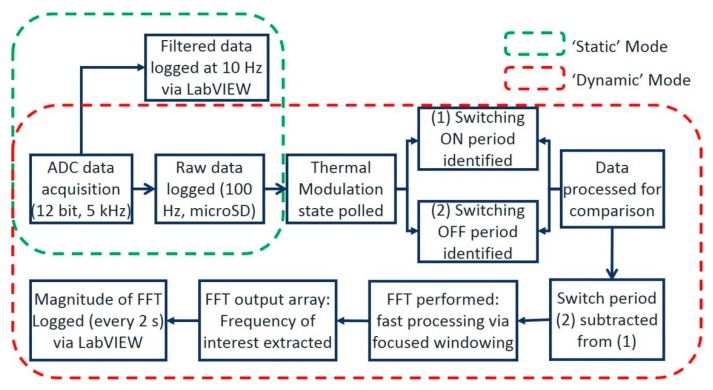
Block diagram of the Teensy microcontroller program under ‘static’ and ‘dynamic’ mode.

Teensy code was written and optimized to perform those steps in real time. To increase the efficiency of the data processing, a reduced select number of samples is processed (128 data points) per cycle. The FFT processing stage is focused on analyzing the frequency range of interest (defined by initial experiments using the MATLAB interface described above). A block diagram is presented below in Figure 6 to show the difference in the code for ‘static’ and ‘dynamic’ operation. The raw sensors’ data were outputted at 100 Hz sampling frequency to store into a microSD card, and the FFT results were also stored while displaying in LabVIEW. 

### 3.2. Results

**Figure 7:**
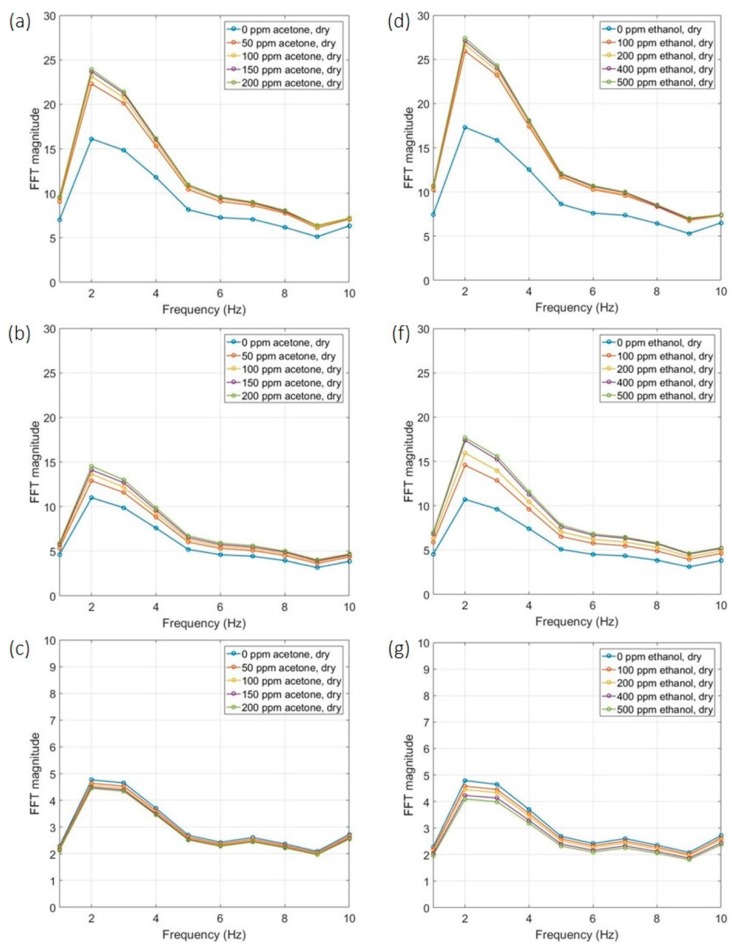
Real-time thermal modulation results of three metal oxide sensors towards acetone (**a**–**c**) and ethanol (**e**–**g**). Response of MOX1 SnO_2_ sensor in (**a**) acetone and (**d**) ethanol; response of MOX2 WO_3_ sensor in (**b**) acetone and (**f**) ethanol; response of MOX3 NiO sensor in (**c**) acetone and (**g**) ethanol.

The ‘dynamic’ operation of sensors was trialed using the same gas testing bench with the same two gases: acetone and ethanol. Five concentration levels were tested for each gas: acetone in 0 ppm, 50 ppm, 100 ppm, 150 ppm and 200 ppm; ethanol in 0 ppm, 100 ppm, 200 ppm, 400 ppm and 500 ppm. Sensors were warmed up for a few minutes prior to the experiment, no need for long stabilization period. Each concentration was trialed for 2 min as this is sufficient to provide concentration information of the target gas. FFT results were presented every 2 s correlates to the 2 s period of the pulse. The magnitude changes to different concentration levels are visualized in LabVIEW. This can be further adapted to be displayed on the handheld device for users. To present the results, the FFT magnitudes averaged over 10 pulses (20 s) were shown in Figure 7. 

**Figure 8:**
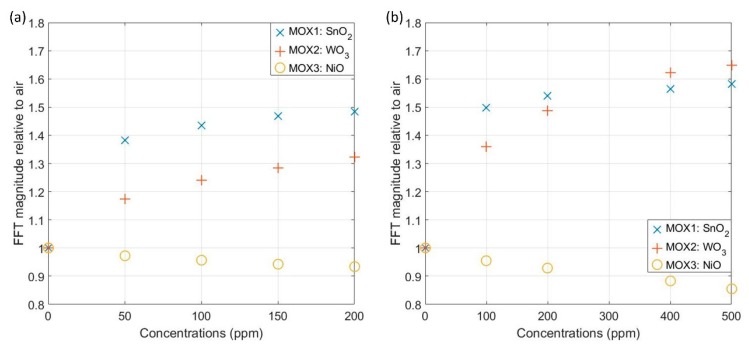
FFT magnitude relative to air results of three sensors: SnO_2_, WO_3_ and NiO in acetone (**a**) and ethanol (**b**).

The FFT output data demonstrate the response to target gas can be represented by the magnitude of the FFT. The peak magnitudes, which occur at 2 Hz, were extracted and the ratio $FFT_{gas}/FFT_{air}$ plotted in Figure 8. Both SnO_2_ and WO_3_ devices magnitude increase with concentration and WO_3_ sensor has greater response towards ethanol. NiO sensor has a smaller response to both gases, which is possible as the NiO sensor is more sensitivity to gases like ammonia [15]. 

## 4. Discussion

The results presented above demonstrate the working mechanism of this novel temperature modulation technique in real-time applications, which is vital for mobile robot application. Based on earlier work [19], the algorithm has now been adapted and optimized for a microcontroller to be used on a gas sensing unit with a sensor array. Three gas sensors can be used at the same time with raw data transmitted at 100 Hz sampling rate and FFT results every 2 s, which can potentially be faster with smaller ‘ON’ and ‘OFF’ period. The sensors, housed inside a robust steel enclosure were tested using a gas testing bench towards acetone and ethanol gas. 

Both ‘static’ and ‘dynamic’ modes were trialed. In ‘static’ DC mode, sensors were operated with a current control circuit and a constant micro-heater temperature at 350 °C. Our MOX sensors show great sensitivities to both gases at low concentration levels with a response speed at 10 s. Although the sensors are superior to other MOX sensors currently available on the market, our dynamic operation mode allows the response time to be further reduced by an order of magnitude, as required for use in diverse mobile robot applications. Due to the nature of MOX sensor, a warming-up period is required before experiments for at least 60 to 90 min, and longer stabilization period (more than 5 mins) is necessary between measurements for a more accurate result. By operating sensors in ‘dynamic’ mode, those imperfections can be compensated. These MOX sensors cannot be installed on a mobile exploration robot when operated in ‘static’ mode, as the robot must be ready for deployment with little or no notice.

**Figure 9:**
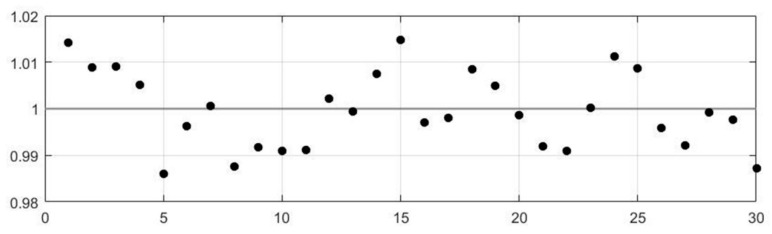
Variance plot of normalized FFT results over 30 datasets.

In ‘dynamic’ mode, the magnitude of FFT results corresponding to the increase of concentration levels. Peak magnitude values were plotted in Figure 8 against background air. The peak frequency for acetone and ethanol were found to be at 2 Hz. It has been previously reported that the frequency peak for CO was created at 1 Hz [13]. Therefore, different peak frequencies in the spectrum can be created with different gases, which is useful to identify gases. In a real-world application, being able to distinguish gases is vital, where in the case of a rescue operation, the gas sensor system should be able to separately identify toxic and explosive gases. The distinct peak frequencies identified for each gas indicate the speed of the chemical reactions occurring in the film layer differs depending on the gas present and in turn, this affects the response of the sensor when a temperature shift is implemented. The experiments were also repeated, and the sensors produced a stable output during the measurements, with any baseline signal drift, existed in ‘static’ operation mode, removed through our signal processing technique. The system is able to operate independently from the baseline, which is critical in a hazardous environment, where it is unlikely a stable baseline will be acquired. Figure 9 shows the variance of normalized FFT values over 30 datasets, which is within ±2% variation.

Different metal oxide coatings respond to gas differently. SnO_2_ coated devices have a high response towards both ethanol and acetone, but a greater response was observed in ethanol. For the same concentration of acetone and ethanol at 200 ppm, SnO_2_ sensor has ~5% more response towards ethanol. WO_3_ sensor also has a smaller response to acetone with a magnitude increase of 30% for 200 ppm acetone, but an increase for 200 ppm of ethanol close to 50%. The NiO sensor has a smaller response towards both gases with less than 1% change at 200 ppm concentration level. The SnO_2_ sensor has an increase close to 38% whilst WO_3_ has a 17% increase for 50 ppm acetone. In 100 ppm ethanol, SnO_2_ and WO_3_ sensors have 50% and 37% increase in response, respectively. This demonstrates our sensor system is capable of low ppm measurements, and further exploration for lower detection limit needed. For a gas in higher concentrations, such as ethanol at 500 ppm, WO_3_ has a slightly larger response of 65% increase compared to SnO_2_ with 60% increase. The visible exponential trend on the plots indicates the devices could get saturated at higher concentrations. These distinctive characteristics of sensors toward different gases can contribute to gas discrimination process alongside the peak frequency changes for a more accurate understanding. 

A Teensy microcontroller is incorporated to drive the circuit and process the data. The output from the microcontroller, which is connected over a USB connection for real-time plotting, can directly show the exact gas present and its concentration (i.e., from the frequency and magnitude of the FFT result). Importantly, this output can directly be used to assess the hazardous nature of the gas detected (from its magnitude) and thus reduces the processing load on the host computer (i.e., for an application involving a mobile robot). This allows the development of a new generation of smart low-cost gas sensors to be used for applications where fast indications (in few seconds or less) are required. The potential applications including, but not limited to, mobile robot, indoor/outdoor air quality monitoring, portable wearable devices, and the internet of things. Our measurement system can also be used alongside an extensive gas mapping and discrimination algorithm, to improve the accuracy and speed notably as compare to using raw sensor data. 

## 5. Conclusions

We have developed an FFT based signal processing algorithms for three temperature modulated resistive metal oxide gas sensors, designed for use on a mobile exploration robot. The three MOX sensors have customized thin film coating of Pt/Pd doped SnO_2_, WO_3_ and NiO, respectively. The sensors are housed inside the gas sensing module with robust steel enclosure for protection. A Teensy 3.6 microcontroller is included inside the module for data processing simultaneously on the sensor array. Algorithms were written and optimized to perform on the microcontroller for real-time data output. Sensors were pulsed between two temperatures, 225 °C and 325 °C for 1 s each. The ‘OFF’ and ‘ON’ data were extracted and FFT was performed. The system was tested with 0 ppm to 200 ppm acetone and 0 ppm to 500 ppm ethanol. The promising results allow the frequency domain to be used for gas detection and potentially to discriminate gases with frequency peaks. This novel technique helps remove baseline drift, which is important in an environment with large temperature variance and reduces the power consumption of the sensors by at least 20%. Furthermore, our technique maintains the fast response of the sensors with response time reduced from 10 s (30 s or more for commercial sensors) to 2 s or less. Operating the sensors in ‘dynamic’ mode mitigates the need for long warm-up periods and stabilization times needed when the devices are operated in ‘static’ mode. Tuning the devices and our data processing systems to operate in our thermal modulation mode has significantly improved the selectivity of the sensors and enables a small array of sensors (three devices) to distinguish a range of gases. The processing technique is now ready for deployment on a mobile robot, and for integration with mapping algorithms. Our data processing algorithms help reduce the complexity of the data output to the robot, thus greatly contributing to our target of producing easy-to-understand information regarding the hazardous environment for the operator. This pre-processed data further reduces the computing load on the host robot computer but enables low concentrations (<50 ppm) of VOCs to be detected in real-time.
